# Comparative Toxicities and Synergism of Apple Orchard Pesticides to *Apis mellifera* (L.) and *Osmia cornifrons* (Radoszkowski)

**DOI:** 10.1371/journal.pone.0072587

**Published:** 2013-09-09

**Authors:** David J. Biddinger, Jacqueline L. Robertson, Chris Mullin, James Frazier, Sara A. Ashcraft, Edwin G. Rajotte, Neelendra K. Joshi, Mace Vaughn

**Affiliations:** 1 Fruit Research and Extension Center, Pennsylvania State University, Biglerville, Pennsylvania, United States of America; 2 USDA Forest Service PSW Station, Albany, California, United States of America; 3 Department of Entomology, Pennsylvania State University, University Park, Pennsylvania, United States of America; 4 The Xerces Society, Portland, Oregon, United States of America; French National Institute for Agricultural Research (INRA), France

## Abstract

The topical toxicities of five commercial grade pesticides commonly sprayed in apple orchards were estimated on adult worker honey bees, *Apis mellifera* (L.) (Hymenoptera: Apidae) and Japanese orchard bees, *Osmia cornifrons* (Radoszkowski) (Hymenoptera: Megachilidae). The pesticides were acetamiprid (Assail 30SG), λ-cyhalothrin (Warrior II), dimethoate (Dimethoate 4EC), phosmet (Imidan 70W), and imidacloprid (Provado 1.6F). At least 5 doses of each chemical, diluted in distilled water, were applied to freshly-eclosed adult bees. Mortality was assessed after 48 hr. Dose-mortality regressions were analyzed by probit analysis to test the hypotheses of parallelism and equality by likelihood ratio tests. For *A. mellifera*, the decreasing order of toxicity at LD_50_ was imidacloprid, λ-cyhalothrin, dimethoate, phosmet, and acetamiprid. For *O. cornifrons*, the decreasing order of toxicity at LD_50_ was dimethoate, λ-cyhalothrin, imidacloprid, acetamiprid, and phosmet. Interaction of imidacloprid or acetamiprid with the fungicide fenbuconazole (Indar 2F) was also tested in a 1∶1 proportion for each species. Estimates of response parameters for each mixture component applied to each species were compared with dose-response data for each mixture in statistical tests of the hypothesis of independent joint action. For each mixture, the interaction of fenbuconazole (a material non-toxic to both species) was significant and positive along the entire line for the pesticide. Our results clearly show that responses of *A. mellifera* cannot be extrapolated to responses of *O.cornifrons*, and that synergism of neonicotinoid insecticides and fungicides occurs using formulated product in mixtures as they are commonly applied in apple orchards.

## Introduction

Pollinator species such as honey bee [*Apis mellifera* (L.) (Hymenoptera: Apidae)] and the Japanese orchard bee [*Osmia cornifrons* (Radoszkowski) (Hymenoptera: Megachilidae)] provide important services in orchard and other agricultural ecosystems [1]–[6]. Members of the genus *Osmia* are important and efficient pollinators of tree fruit crops such as apples, plums and cherries, among other economically important fruit and nut crops [7]–[9]. The Japanese orchard bee (also known as the Japanese horn-faced bee) is commercially used for pollination of pears and apples in Japan [9]. This species was introduced into the mid-Atlantic region of the United States by USDA scientists in 1977 [10], [11], has since become commercially available, and is now an important (and efficient) wild pollinator in tree fruit orchards in Pennsylvania [12], [13]. One *O. cornifrons* can set up to 80% more apple flowers per day compared with flowers set by one honey bee, *A. mellifera* (L.), worker [14]. These two species are complementary pollinators of apple, peach and pear orchards in the Northeastern United States [12], [13], [15]–[19].

A recent significant decline in bee populations in the United States [20]–[23] and elsewhere [24] has led researchers to investigate the effects of possible factors such as pesticide residues [25]–[33], different types of pathogens [34]–[36], adjuvants [37], various parasites [38], floral resource availability and diversity [3], and pest management and agricultural practices [39] on the health, abundance and diversity of different species of bees and pollinators. Although most past research has emphasized the effects of these factors on either *A. mellifera* or on bumble bee species, the relative effects of pesticide mixtures (e.g., insecticides and fungicides) on two different species of pollinators (for instance, *O. cornifrons* versus *A. mellifera*) have not been investigated.

In agricultural production systems, various classes of chemicals are used for management of various pests and diseases. Commercial and farmer tank-mixes of insecticide and fungicide are used to reduce operational or production costs (or both). In tree fruit orchards in Pennsylvania, pesticides are applied for management of a complex of diverse pest species. These include fruit-feeding Lepidoptera, leaf rollers, mites, aphids, plum curculio and stink bugs [40]–[42]. The most critical period to reduce pollinators' exposure to pesticide application in apple orchards is bloom. Different pesticides are applied for control of pests such as rosy apple aphid, European apple sawfly, and plum curculio just before, during, and immediately after bloom. This timing is also critical for control of fungal diseases such as apple scab, *Venturia inequalis* (Cooke) and apple powdery mildew, *Podosphaera leucotricha* (Ell. & Evherh.) [40]. However, information about the toxicity of these toxicant mixtures to beneficial invertebrates including pollinators such as *O. cornifrons* and *A. mellifera* must be obtained if rational conservation plans for pollinators are to be implemented. Well-designed laboratory bioassays provide the scientific basis for such decisions.

Our studies were done to provide basic information about the comparative toxicities of commonly-used orchard pesticides and pesticide-fungicide mixtures to *O. cornifrons* and *A. mellifera*. Results of our experiments were used to test the general premise that one species such as *A. mellifera* can serve as a surrogate species for a larger taxonomic group such as several families of bees, or as a surrogate for an arbitrary group such as all terrestrial arthropods [43]. Our study expands a previous investigation of differential toxicity of imidacloprid to *A. mellifera* versus *Bombyx mori* [44] and suggests the direction in which the study of ecotoxiciology of pollinators can progress [45]. Of most importance, our study provides an example of the type of information necessary to improve the sensitivity of testing pesticides on diverse species in the superfamily Apoidea [46] in the future.

## Materials and Methods

### Insects

*O.cornifrons* were purchased from a single source in Wisconsin where they had been reared in an organic apple orchard. Because fruit from this orchard were used only for cider production, pesticide use was minimal. Larvae were reared in natural *Phragmites* reed bundles and in wooden blocks lined with paper straws. Cocoons containing the overwintering adults were removed and refrigerated at 3°C until 1 April to ensure that their chilling requirements had been met. Loose cocoons were then held inside an incubator (25°C, constant darkness) until adults emerged. Adults were held in darkness until treated 24–72 hr after emergence (24 hr for the males; 24–72 hr for the females). Emergence occurred from April through May. *A. mellifera* used in this study were purchased as new packages from Gardner Apiaries, Spell Bee (Baxley, GA). Each package included a queen and workers. The packages were exposed to the miticides fluvalinate and coumaphos for bee mite control. They were then put into hives pre-sterilized by irradiation. Colonies were established in the spring and kept in an isolated area at least 6 km from any pesticide applications.

### Treatments

For treatment, cages were made of a Petri dish (100×20 mm) encasing a 100 mm-long wire mesh cylinder constructed of hardware cloth (with 3×3 mm openings). One side of the Petri dish had two holes made with a heated cork borer. The larger hole was used to put treated bees into the dish. Once the cage was full, the hole was sealed with tape. The other (smaller) hole held a glass vial for *ad lib* feeding of a 50% sucrose solution.

For each replication (per chemical or combination) with a species, 10 bees were placed in a 50 ml centrifuge tube with a pair of forceps. The tube was placed in an ice bath for 1–2 min. to immobilize the bees, which were then poured onto a paper towel. If a bee did not move after the cold anesthesia, it was discarded from the bioassay. Each bee was picked up at the base of the wings, treated on the thorax, and placed in the cage. One µl/bee was applied with a Hamilton repeating dispenser (Hamilton Company, Reno, NV) that held a 50 µl syringe. After each group of 10 bees was treated, the cage was set aside and checked after ∼3 min. to ensure that all bees appeared to be healthy. Six cages were placed on their sides inside a plastic container, which also contained a moist paper towel and a jar of a saturated NaCl solution to maintain ∼75% RH. Six caps from 20 ml scintillation vials were used to separate and hold the cages in place.

Water was used as the solvent to mimic what growers use in the field to apply the pesticides. No problems with formulation solubility were observed. The commercial formulations (AI% ; manufacturer) were Assail 30SG (acetamiprid 30%; United Phosphorous Inc., King of Prussia, PA), Dimethoate 4EC (dimethoate 43.5%; Drexel Chemical Company, Memphis, TN), Imidan 70W (phosmet 70%; Gowan Company, Yuma, AZ), Provado 1.6F (imidacloprid 17.4%; Bayer CropScience, Research Triangle Park, NC) and Warrior II (λ - cyhalothrin 22.8%; Syngenta, Wilmington, DE). Interactions of the fungicide Indar 2F (fenbuconazole 22.86%; Dow AgroSciences LLD, Indianapolis, IN) in a 1∶1 proportion with Assail 30SG and in a 1∶2 proportion with Provado 1.6F were also tested.

### Experimental Designs and Data Analyses

In all experiments, mortality was tallied after 48 hr. Control mortality was <5%. Average control mortality was 2.7%. At least 6 doses of each pesticide plus a control (water only) were tested in each replication per pesticide per bee species. Each replication included a total of 60–135 bees of each species depending on species' availability. Dose-mortality regressions were estimated assuming the normal distribution (i.e., probit model) with the computer program PoloPlus [47] as described by Robertson et al. [48]. We used a two-step procedure to analyze data for each chemical. In the first step, we examined plots of standardized residuals for outliers, which were then eliminated from the data sets. The second and final probit analysis was done to test hypotheses of parallelism (slopes not significantly different) and equality (slopes and intercepts not significantly different) with likelihood ratio tests [48]. PoloPlus also calculated lethal dose ratios (LDR's) of the most toxic chemical compared with all other chemicals for each species. An LDR provides a means to test whether two LD's are significantly different (i.e., when the 95% CI for the LDR did not include the value 1.0 [47], [48]).

For tests with a mixture, at least 5 doses of the mixture that bracketed 5–95% mortality were tested concurrently with experiments with at least 5 doses of individual mixture components. As before, 60–135 bees of each species were tested depending on species' availability. To test the hypothesis of independent joint action of fenbuconazole with acetamiprid or imidacloprid, we used the computer program PoloMix [49]. Assuming independent joint action of two mixture chemicals, test subjects can die of three possible causes. The first cause is natural mortality, with a probability *p*_o_ (a constant). The other two causes of mortality are the probabilities of mortalities for chemical 1 or chemical 2. For the first chemical, the probability of response (*p*1) is a function of dose *D*1. Usually, the probit or logit of dose *X* of chemical 1 is log(*D*1) (i.e., *X*1 = log[*D*1]). For the second chemical, the probability of response (*p*2) is a function of dose *D*2. If these three causes of mortality are independent, the probability of death (*p*) is *p* = *p*0+(1−*p*0)*p*1+(1−*p*0)(1−*p*1) *p*2. When each “+” sign means “or” and each product means “and,” this equation means that the total probability of death equals death from natural causes (*p*0), or no death from natural causes (1−*p*0) and death from the first chemical [e.g., (1−*p*0)*p*1], or no death from natural causes or from the first chemical [i.e., (1−*p*0)(1−*p*1)], but death from the second chemical [i.e., (1−*p*0)(1−*p*1) *p*2]. The χ2 statistic produced by PoloMix [49] was used to test the hypothesis of independent joint action. This test statistic is calculated by obtaining an estimate for the probability of mortality (*p*) for several dose levels of the two components and then comparing 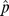 (the estimate of *p*) with the observed proportion killed at the corresponding dose levels. The three contributions to *p* are estimated separately. First, *p0* is calculated as the proportional mortality observed in the control group. Next, *p1* and *p2* are estimated from bioassays of chemical 1 and chemical 2, with test statistics estimated from PoloPlus [47].

## Results and Discussion

### Responses of *O. cornifrons* (Table S1)

The hypotheses of parallelism and equality of response of *O. cornifrons* to the five pesticides were both rejected (*P* = 0.05). At LD_50_ (the most reliable point of comparison for dose-response regressions [48]), the decreasing order of toxicity was dimethoate>λ-cyhalothrin>imidacloprid>acetamiprid>phosmet. LDR50's indicated three groups of significantly increasing toxicity. The least toxic group consisted of phosmet, acetamiprid and imidacloprid. λ-cyhalothrin was significantly more toxic than the first group, and the most toxic pesticide, dimethoate, was ∼70 times more toxic than phosmet (the least toxic pesticide). At LD90, the increasing order of toxicity was dimethoate>λ-cyhalothrin>phosmet>imidacloprid>acetamiprid. Responses to acetamiprid and imidacloprid were not significantly different at LD_90._ Significantly increased susceptibilities to phosmet and λ- cyhalothrin were indicated by their LDR90's. Dimethoate was ∼60 times more toxic than acetamiprid.

### Responses of *A.mellifera* (Table S1)

The pattern of responses for *A.mellifera* differed considerably from that of *O. cornifrons*. For *A.mellifera*, responses were also neither parallel nor equal (*P* = 0.05). The decreasing order of toxicity at LD_50_ was imidacloprid>λ-cyhalothrin = dimethoate>phosmet>acetamiprid. LDR50's indicated two levels of decreasing toxicity relative to imidacloprid: λ-cyhalothrin or dimethoate <phosmet. At LD_90_, the increasing order of toxicity was dimethoate>imidacloprid>λ- cyhalothrin>phosmet>acetamiprid. Dimethoate was ∼1200 times more toxic than acetamiprid as this response level. LDR90's suggested three groups of response: dimethoate>λ- cyhalothrin or imidacloprid>phosmet>acetamiprid.

### Comparative Responses of the Pollinator Species (Table S1)

Neither species was consistently more susceptible than the other. Their responses to the two neonicotinoid pesticides were parallel, but not equal. *O. cornifrons* was significantly more susceptible than *A.mellifera* to acetamiprid at LD_50_ (*i.e.*, the LDR_50_ did not bracket the value 1.0 [47], [48]). At LD_50_, *O. cornifrons* ∼12 times more susceptible than *A.mellifera*. In contrast, *A.mellifera* was significantly more susceptible to imidacloprid at the 50% response level (LDR50 did not bracket the value 1.0). At LD_50_, *A.mellifera* were ∼26 times more susceptible than *O. cornifrons*. Responses to the two organophosporous pesticides — dimethoate and phosmet — were also not consistent by species. The hypotheses of parallelism and equality were each rejected in tests with these chemicals. For dimethoate, *O. cornifrons* was 3.7 times more susceptible than honey bees at LD_50_, but the regression slope (7.62) was so steep for *A.mellifera* that relative toxicities were reversed at the LD_90_ (at 90% response, honey bees were 3.2 times more susceptible that *O. cornifrons*). With phosmet, the slope of the regression line for *A.mellifera* was very shallow and its line crossed the dose-response line for *O. cornifrons* at the upper end of response. At LD_50_, *A.mellifera* workers were ∼3 times more susceptible than *O. cornifrons*, but at LD_90_, *O. cornifrons* was ∼2 times more susceptible than *A.mellifera*.

Responses to the only pyrethroid tested, λ- cyhalothrin, were neither parallel nor equal. At LD_50_, *A. mellifera* was 3 times more susceptible than *O. cornifrons*. LD90's for the two species were not significantly different.

### Responses to Mixtures (Table 1, Figure 1)

**Table 1:** Responses of *Osmia cornifrons* and *A. mellifera* to to acetamiprid mixed with indar.

Chemical	Mixture	Species^a^	n	Slope ± S.E.	LD_50_ b	95% CL	LDR^c^	95% CI*
Acetamiprid	only	A	245	1.39±0.41	64.6	38.1–252	1.0	–
Indar	only	A	330	Not toxic				
Acetamiprid	Indar (1∶1)	A	360	3.13±0.53	14.3	8.5–30.8	4.5	2.5–8.2
Acetamiprid	only	O	272	0.97±0.18	4.0	1.1–7.1	1.0	–
Indar	only	O	60	Not toxic				
Acetamiprid	Indar (1∶1)	O	99	1.94±0.42	2.1	1.1–3.2	1.9	0.95–3.9
Imidicloprid	only	A	310	1.26±0.15	0.2	0.1–0.3	1.7	0.92–3.1
Indar	only	A	330	Not toxic				
Imidicloprid	Indar (2∶1)	A	300	1.84±0.33	0.3	0.1–0.4	1.0	–
imidacloprid	only	O	310	1.26±0.15	3.8	1.7–12.6	1.7	0.8–3.7
Indar	only	O	90	Not toxic				
Imidicloprid	Indar (2∶1)	O	522	3.11±1.09	6.6	1.4–9.6	1.0	–

a A is *A.mellifera*, O is *Osmia cornifrons*.

b LD is expressed as µg/bee.

c LDR is higher LD÷lower LD.

* If the 95% CI of the LDR includes the value 1.0, the LD's are not significantly different.

**Figure 1:**
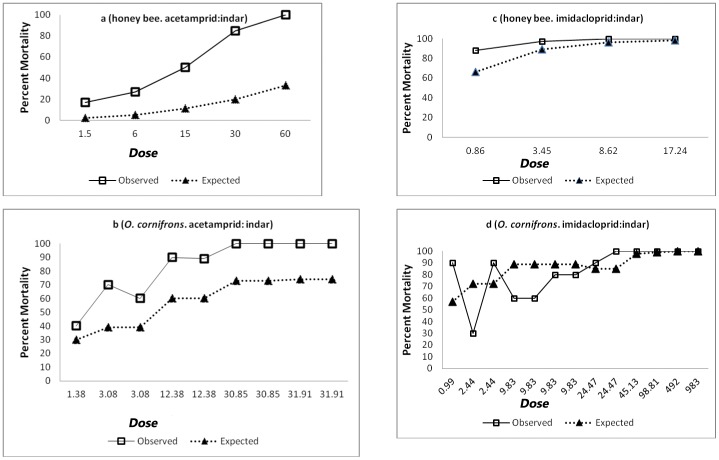
(a–d). Effects of mixtures on *O. cornifrons* and *A. mellifera*. Boxes are responses observed and triangles are responses predicted assuming the model of independent joint action (see text). Dose is in units of µg/bee. Figure 1a is for *A. mellifera* treated with acetamiprid∶indar; Figure 1b is for *O. cornifrons* treated with acetamiprid∶indar; Figure 1c is for *A. mellifera* treated with imidacloprid∶indar; Fig. 1d is for *O. cornifrons* treated with imidacloprid∶indar.

Fenbuconazole was minimally toxic to both species. In combination with acetamiprid, the 1∶1 mixture was ∼5 times more toxic than acetamiprid alone to *A. mellifera* at LD_50_ (Fig. 1a). The toxicity of the mixture was 2 times greater than acetamiprid to *O. cornifrons* at LD_50_ (Fig. 1b). Fenbuconazole enhanced responses of imidacloprid slightly, but significantly, for both species. Although responses at the LD_50_ were not significantly different, a greater effect was apparent at higher levels of response (Fig. 1c, d). χ2 values of observed vs. expected values were small but still significant. Rejection of the null hypothesis of independent joint action of the fenbuconazole with either of the two pesticides indicated that significant synergism occurred in both bee species. In contrast, another neonicotinoid – thiacloprid – applied as technical material in 100% ethanol, was synergized 1141- and 559-fold by the addition, respectively, of the fungicides triflumizole and propiconazole, which are in the same class as fenbuconazole [49].

## Conclusions

Because of their highly controlled conditions and rigorous experimental design, laboratory bioassays provide the ideal means to estimate comparative responses of *A. mellifera* and wild bees to pesticides. Our results clearly show that the response of *A. mellifera* cannot be extrapolated to the response of *O.cornifrons*. Such results might be expected given the extensive body of information from analogous (and as rigorously designed and statistically analyzed) experiments showing that responses of the Lepidopteran families Tortricidae and Lymantriidae vary significantly among genera, within a single genus, among populations or even among sibling groups of the same genetic strain [47], [50], [51] tested with the same pesticide. Equally rigorously designed bioassays among Apoidea populations of the same species, species of the same genus, and among genera done to evaluate variation at all levels of response are clearly needed. Natural variation in response for a populations of single species to single pesticides, whether applied as pure active ingredient or formulated material, also needs to be estimated [52]. This systematic approach should be part of the overhaul of the pesticide registration process as suggested by Decourtye et al. [46]. Finally, without direct access to raw data, we have no way to compare our results statistically with previously published information from previous experiments with *A. mellifera* tested with neonicotinoid insecticides [53], let alone explain why these differences occurred. Until such basic comparisons are made possible even for one species such as *A. mellifera*, bee toxicologists and ecologists will continue to debate possible explanations for different contact and oral LD_50_'s. One solution to this situation would be establishment of a free-access network for raw data from all experiments that have been used in applications for pesticide registrations by the US EPA (US EPA) and the European Union (EU).

Our results further suggest that the current practice of using *A. mellifera* as a surrogate species for >90,000 other species of non-target insects, including *O. cornifrons*, in the pesticide registration process as required by the United States EPA is scientifically flawed, and needs significant modifications. Inherent in this registration process is the assumption that the response of *A. mellifera* can be extrapolated to responses of the many different species of predatory and parasitic arthropods that are important to successful Integrated Pest Management (IPM) programs, but which belong to several distantly related orders. This assumption has been shown to be untenable in other studies: many pesticides labeled as “reduced risk” or “organophosphate replacements” by the United States EPA and that have used toxicity tests with *A. mellifera* as part of the criteria for these classifications are in fact toxic to non-target insects and disruptive to IPM programs in tree fruit crops [42], [52], [54], [55]. Use of *A. mellifera* as a surrogate species has also been questioned by members of the European

Union [56], which also currently requires toxicity data for multiple insect species [43]. According to Decourtye et al. [46], 42 studies report deleterious side effects on other bee species despite the fact they passed risk assessment on honey bees. Considering the fact that pollinator species significantly differ in their relative toxicity to pesticides, the United States EPA needs to review its current policy on pollinator toxicity that requires data from contact toxicity (from pesticide residues) studies designed to investigate acute effects of a pesticide chemical (under the registration process) on individual bees [57], in addition to adding studies of sublethal effects to these toxicants [58], [59].

## Supporting Information

Table S1 **Responses of** ***O. cornifrons*** **and** ***A. mellifera*** **to pesticides commonly used in apple orchards (complete regression results).**
(DOC)
