# Supplementary material for: Comparative Toxicities and Synergism of Apple Orchard Pesticides to Apis mellifera (L.) and Osmia cornifrons (Radoszkowski)
Source: PLoS One. 2013 Sep 9;8(9):e72587. doi: 10.1371/journal.pone.0072587 (PMC3767698; doi:10.1371/journal.pone.0072587)
Supplement: Table S1 — Responses of O. cornifrons and A. mellifera to pesticides commonly used in apple orchards (complete regression results). (DOC) [file pone.0072587.s001.doc]

Table S1. Responses of *O. cornifrons* and *A. mellifera* to pesticides commonly used in apple orchards (complete regression results)

| Chemical | Formulation | Species^a^ | n | Slope ± S.E. | het^b^ | LD_50_^c^ | 95% CL | LDR^d^ | 95% CI* | LD_90_^c^ | 95% CL | LDR^d^ | 95% CI* | P^e^ | P^f^ |
| --- | --- | --- | --- | --- | --- | --- | --- | --- | --- | --- | --- | --- | --- | --- | --- |
|  |  |  |  |  |  |  |  |  |  |  | 172 - |  |  |  |  |
| acetamiprid | Assail | A | 245 | 1.39 ± 0.41 | 1.2 | 64.6 | 38.1 - 252 | 1.0 | – | 537 | >1000 | 1.0 | – |  |  |
|  |  | O | 242 | 1.05 ± 0.18 | 1.9 | 5.2 | 2.1 - 9.0 | 12.3 | 6.1 - 25.0 | 87.1 | 37.9 - 719 | 6.2 | 1.0 -37.4 | P | NE |
| dimethoate | dimethoate | A | 450 | 7.62 ± 1.16 | 1.0 | 0.31 | 0.28 -0.34 | 1.0 | – | 0.46 | 0.41 - 0.54 | 3.2 | 1.2 -8.1 |  |  |
|  |  | O | 156 | 1.04 ± 0.19 | 2.4 | 0.09 | 0.02 - 0.20 | 3.7 | 2.0 - 6.6 | 1.43 | 0.49 - 50 | 1.0 | – | NP | NE |
| phosmet | imidan | A | 250 | 1.09 ± 0.18 | 3.2 | 1.9 | 0.91 - 11.2 | 3.3 | 1.9 - 5.5 | 27.5 | 6.2 - 6300 | 1.0 | – |  |  |
|  |  | O | 254 | 3.36 ± 0.48 | 3.7 | 6.1 | 4.0 - 8.9 | 1.0 | – | 14.6 | 9.7 - 50.0 | 1.9 | 0.5 – 6.8* | NP | NE |
| imidacloprid | Provado | A | 310 | 1.26 ± 0.15 | 3.1 | 0.15 | 0.05- 0.32 | 26.1 | 14- 49 | 1.53 | 0.63 -10.9 | 37.8 | 12.1 -118 |  |  |
|  |  | O | 522 | 1.09 ± 0.15 | 4.0 | 3.82 | 1.65 -12.6 | 1.0 | – | 57.7 | 16 - 1230 | 1.0 | – | P | NE |
| λ-cyhalothrin | Warrior | A | 360 | 1.22 ± 0.11 | 6.8 | 0.30 | 0.08 - 1.57 | 3.0 | 2.0 - 4.5 | 3.4 | 0.08 - 657 | 1.5 | 0.7 - 3.3* |  |  |
|  |  | O | 466 | 1.69 ± 0.23 | 0.8 | 0.91 | 0.69 - 1.17 | 1.0 | – | 5.2 | 3.48 - 9.94 | 1.0 | – | NP | NE |

^a^ A is *A.mellifera*, O is *Osmia cornifrons*.

^b^ het is the heterogeneity adjustment factor.

^c^ LD is expressed as µg/bee.

^d^ is higher LD ÷ lower LD.

^e^ P means that the hypothesis of regression slopes (same slopes) could not be rejected at *P* = 0.05. NP means that the hypothesis of regression slopes was rejected at *P* = 0.05 [47, 48].

^f^ NE means that the hypothesis of equal lines (same slopes, same intercepts) could not be rejected at *P* = 0.05 [47, 48].

*If the 95% CI of the LDR includes the value 1.0, the LD’s are not significantly different [47, 48].
